# Medical Qigong for Mobility and Balance Self-Confidence in Older Adults

**DOI:** 10.3389/fmed.2020.00422

**Published:** 2020-08-14

**Authors:** James E. Stahl, Shoshana S. Belisle, Wenyan Zhao

**Affiliations:** ^1^Dartmouth-Hitchcock Medical Center, Section General Internal Medicine, Lebanon, NH, United States; ^2^The Dartmouth Institute for Health Policy & Clinical Practice, Lebanon, NH, United States; ^3^The Geisel School of Medicine at Dartmouth, Hanover, NH, United States; ^4^The Dartmouth Synergy Clinical and Translational Science Institute, Biostatistics Consultation Core, Lebanon, NH, United States

**Keywords:** falls, stability, balance, mind-body medicine, health confidence, qigong

## Abstract

**Background/Objectives:** Physical inactivity, sedentary lifestyle, and impaired neuromuscular function increases fall risk and fractures in our aging population. Mind-body modalities, improve strength, balance and coordination, mitigating these risks. This study examined whether a manualized Medical Qigong protocol measurably improves balance, gait, and health self-confidence among older adults.

**Design:** Randomized prospective cohort pre-post study with wait time control.

**Setting:** Two martial arts centers in Massachusetts and Arizona.

**Participants:** Ninety-five adults age ≥ 50 (mean age 68.6 y.o., range 51–96) were randomly assigned to an immediate start group (N = 53) or 4-week delayed start group (*N* = 43).

**Intervention:** A 10 form qigong protocol taught over 12 weekly classes.

**Measurments:** Primary outcome measures were the Community Balance and Mobility Scale (CBMS) and Activities-Specific Balance Confidence (ABC) Scale. Data was collected at baseline, 1-month and 4-months.

**Results:** Both groups at both sites demonstrated improved balance and gait (CBMS + 11.9 points, *p* < 0.001). This effect was strongest in patients in their 60 s (CBMS +12.9 *p* < 0.01) and 70 s (CBMS + 14.3, *p* < 0.001), was equal across genders and socioeconomic status. Balance self-confidence did not significantly change (ABC + 0.9, *p* = 0.48), though several elements within ABC trended toward improvement [e.g., walk up/down ramp (*p* = 0.07), bend over/pick up (*p* = 0.09)]. Falls in the past year was inversely correlated with balance self-confidence (*p* = 0.01).

**Conclusion:** A 12-week manualized Medical Qigong protocol significantly improved balance and gait and modestly improved balance self-confidence among older adults. Medical Qigong may be a useful clinical intervention for older adults at heightened risk for falls and related injuries.

**Clinical Trial Registration:** www.ClinicalTrials.gov, identifier: NCT04430751.

## Introduction

Balance is a complex phenomenon, involving the ability to control the body's equilibrium in both static and dynamic situations. It is the result of a complex interaction between external and internal sensory systems, reactive and proactive systems, anticipatory mechanisms, and an active internal representation of the body schema (1). When this system fails, we fall.

The cost of falling is large and growing. In the United States, as adults live longer, falls and related injuries, such as fractures and head trauma, have increasingly become a significant public health concern. Approximately one in four older adults (age 65 and older) fall each year, making falls the leading cause of fatal and non-fatal injuries in this growing population (2). Of the estimated 29 million falls in 2014, over 7 million required medical attention (2). Twenty percent of falls result in serious fractures or brain injury (3, 4). In 2016, 29,668 U.S. older adults died from falls, a 31% increase in annual fall-related deaths compared to 2007 (5). In addition to the high personal cost, there is considerable societal cost. In 2015, total medical costs from fatal and non-fatal falls exceeded $50 billion (6). The cost of fatal falls alone was estimated to be $745 million (6). Given that the older adult population is expected to climb from 49.2 million, in 2016, to 78 million in 2035 (7), projected falls, injuries, and their associated costs are also expected to rise; unless major public health interventions are implemented. It is therefore essential to find effective, safe, and cost-effective strategies to help reduce falls and related injuries among older adults.

Risk factors for falling can be considered intrinsic and extrinsic, and modifiable and unmodifiable. The greatest predictors for falling include impaired balance, polypharmacy (concurrent use of multiple medications often for the same illness), and history of previous falls (8). Intrinsic modifiable factors include muscle weakness, gait and balance problems, and fear of falling. Extrinsic modifiable factors include polypharmacy and physical hazards in home. Unmodifiable factors include age and history of prior falls. Many interventions have been tried to mitigate this problem (9). These are usually categorized as either single focused interventions (10), focusing on a particular form of training, or multifactorial interventions, focusing on both intrinsic and extrinsic risks (11). The most effective and resource intensive interventions to date have been multifactorial ones that include over 50 h of training which feature complex and challenging balance exercises (12).

The National Council on Aging currently recommends balance-focused exercise as the primary strategy for preventing falls (13). Appropriate exercise programs for older adults, especially those with osteoporosis or previous fractures, must take into account safety and accessibility (14). Movement practices, such as yoga, Tai Chi and Qigong, can be tailored to the fitness capacities of older adults, and may be a useful alternative to conventional exercise programs. Practices such as Tai Chi and Qigong, that combine gait, balance, muscle strengthening, coordination and functional exercises, seem to have the greatest impact on balance among older adults (15). Additionally, these physical exercises reduce risk of osteoporosis and increase fitness and bone density, thereby potentially reducing the severity of falls if and when they do occur (16).

Qigong is an ancient Chinese meditative movement practice that integrates postures, meditation, visualization, and breathing to promote strength, flexibility, balance, mindful awareness, spiritual development, and relaxation (17). Qigong is the foundation upon which Tai Chi and many other East Asian practices are based. The practice is an integral part of both ancient and modern Chinese medical practice, and is now a popular mind-body wellness technique in the United States as well. An estimated 2.9 million US adults practiced either Tai Chi, Qigong or both in 2012 (18). These tools are well-suited to the falls problem in that they address all the main aspects of the equilibrium system. They develop and train the sensory systems involved in balance and sense of position and state. They develop static and dynamic strength. They develop situational awareness, mindfulness and reactive strategies for disequilibrium and anticipatory strategies for movement.

Tai Chi, which has roots in Qigong, has been widely studied for its capacity to promote balance and mobility in older adults; a review of 20 years of research concluded that Tai Chi offers substantial health benefits, including improved balance (15, 19). Tai Chi and Qigong training have already shown promise in retarding bone loss in postmenopausal women and for improving balance and balance self-confidence among breast cancer survivors (20–22). Qigong has been shown in systematic review to help Quality of Life, sleep balance, strength, trunk flexibility and improved cardiovascular conditioning (23) as well as cognitive function and depression, factors influencing balance and confidence. Unfortunately, Tai Chi for many is considered complex and challenging to learn. Medical Qigong's forms are typically simpler (17). In addition, the tools taught in previous studies on Tai Chi and Qigong vary substantially in content and emphasis and as a result suffer from problems with reproducibility, consistency and accessibility.

The current study involves the use of a Bagua-based medical Qigong protocol that has been manualized for reproducibility and consistency as a primary strategy to improve balance, gait, and health self-confidence. Bagua is one of the three main internal martial arts (Taijiquan, Baguazhang, Xingyiquan) and emphasizes circular and spiraling movements. The current protocol is called Golden Qi Ball, and was developed by Qigong Master Michael Leone. It includes 10 Qigong forms, designed to improve balance and flexibility and to be easily mastered by older adults.

We hypothesize that medical qigong can improve both physical function and balance self-confidence. The purpose of this study therefore is to prospectively evaluate and objectively measure the effect of the mind-body intervention of a manualized Qigong form on intrinsic risk factors for falling, specifically, mobility, balance and fear mobility, balance and fear of falling, as measured by balance health confidence.

## Methods

This multi-site study examined the effects of a manualized medical Qigong protocol on balance and mobility and balance self-confidence.

### Study Design

This was a prospective cohort pre-post intervention study with a randomized wait time control. The study took place in two martial arts training centers in the United States: Body Mind Systems Martial Arts Center in Swampscott, Massachusetts, and Sun City West Zen Wellness Center in Sun City West, Arizona. The center directors acted as study site directors and were trained to use the evaluative instruments. Staff implemented a manualized training protocol called “Golden Qi Ball.” Site Directors were responsible for participant recruitment and enrollment, evaluations and collections of study measures, as well as the weekly manualized Qigong instruction. The study was approved by the Committee for the Protection of Human Subjects at Dartmouth Hitchcock Medical Center and Dartmouth College. All participants provided written informed consent.

### Participant Recruitment, Selection, and Inclusion and Exclusion Criteria

**Figure 1:**
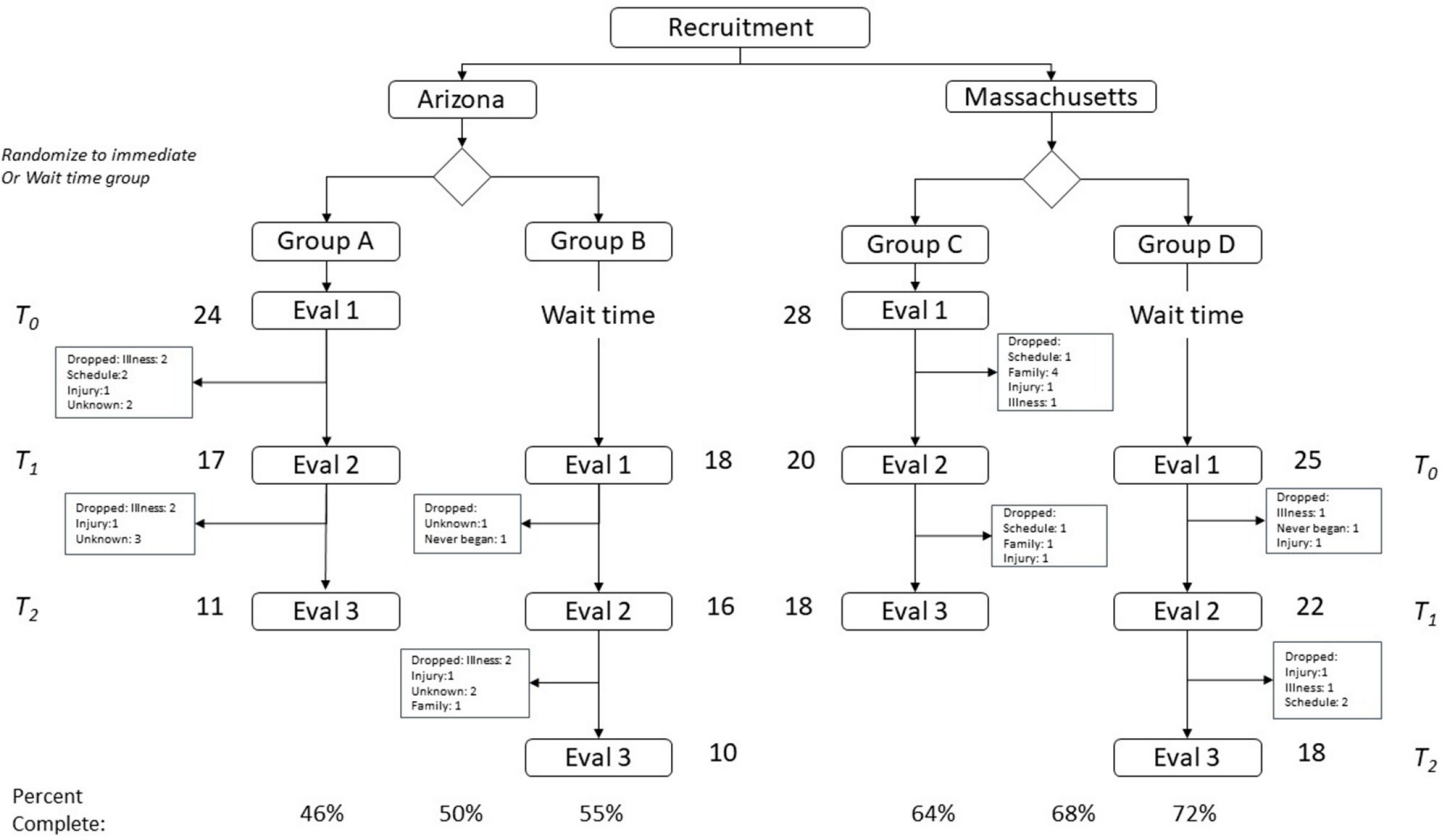
Change in subjects balance confidence from pre-intervention to post-intervention.

Recruitment occurred through advertisements in the local newspapers. Adults 50 years of age or older who presented at the two locations (Massachusetts, Arizona) were considered eligible for participation. They were excluded if they could not give informed consent. To allow for controlled analysis, on presentation at each site, participants were randomly assigned to one of two cohorts: (1) immediate start (A) and (2) delayed start (B). The immediate arm began at the next available class. The delayed start group began 4 weeks later. During their wait, this group was offered lectures on history and practice of traditional Chinese medicine and classes on basic stretching (Figure 1).

### Inclusion/Exclusion Criteria

Subjects had to be adults > 50 years-old. Subjects were excluded if they could not give informed consent.

### Description of Intervention

**Figure 2:**
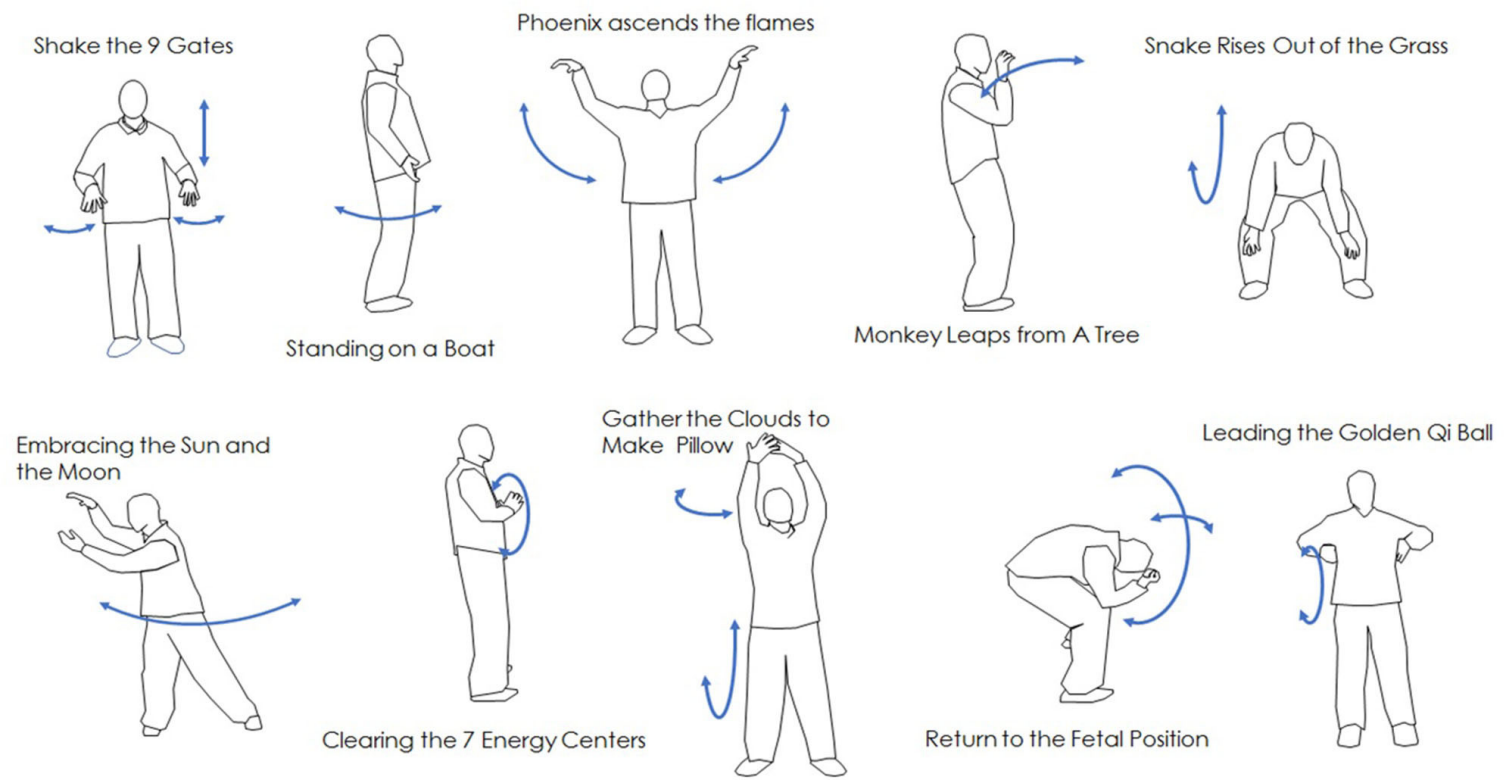
Flow chart of subject recruitment, participation and retention.

The study intervention consisted of a 12-week training program that introduced students to a progressive series of 10 Qigong “forms” that are designed to build upon each other to restore balance and function and to enhance well-being. These forms involve both physical movement and visualization. The 10 Qigong forms included in the protocol are illustrated in Figure 2.

Classes were each ~1 h long and were offered in a group format with ~10–20 students per class. Participants were asked to attend at least one class per week though they could attend more if desired. Attendance was collected in order to be able to track adherence, dose effect and potential reasons for drop out.

The teachers were all certified instructors in the same Bagua-based medical Qigong protocol. They had completed a minimum 200-h training program in teaching medical Qigong and had passed a certifying exam in medical Qigong.

### Outcome Measures

Demographic data was collected during enrollment and included questions about potential confounding factors such as prior martial arts experience or gait training. The outcome measures collected were: the Activities-specific Balance Confidence Scale (ABC) and the Community Balance and Mobility Scale (CBMS).

All instructors received training in the application of the ABC and CBMS measurement instruments.

#### Community Balance and Mobility Scale

The Community Balance and Mobility Scale (CBMS) is a performance-based measure designed to evaluate balance and mobility in ambulatory individuals in the community setting (24). The instrument consists of 13 items using a 6-point rating scale. Six of the items are performed on both right and left sides, bringing the item total to 19 tasks. Possible scores range from 0 to 96, and higher scores indicate better balance and mobility. A strength of the CBMS is that it avoids the ceiling effect observed in other gait and balance assessments for community-dwelling older adults and allows measurement of function from the frail to the healthy (25). The tool takes ~20 min to perform and must be conducted by a trained observer who scores the measurements. For this particular study, the site directors and selected staff members at the study sites were trained to administer the CBMS.

Inter-rater reliability was ensured by randomly sampling individuals during the testing, video recording their CBMS test, de-identifying the recording, and having evaluators at the other site independently and anonymously score the videos. None of the evaluators had any contact or had access to any identifying information on the subjects at the other site. All videos were erased after they were scored. Inter-rater reliability was estimated by Cohen's Kappa.

#### Activities-Specific Balance Confidence Scale

The Activities-specific Balance Confidence (ABC) Scale was designed to assess balance confidence among older adults (26, 27). It is a validated self-report psychometric instrument that consists of 16 items that are rated on a scale from 0 to 100% regarding degree of confidence associated with a particular activity. It takes ~15 min to complete.

### Timing of Evaluations

Subjects completed the evaluations at three points throughout the course of the study: at *T* = 0. (prior to starting classes); *T* = 1 (1 month after starting classes); *T* = 3 (following completion of 12 weeks of classes). The wait-time subjects began their evaluations and instruction in Qigong 1 month after the immediate-start group. They completed evaluations at the same time intervals.

### Statistical Analyses

The blinded data was submitted to Dartmouth Synergy Clinical and Translational Science Institute, Biostatistics Consultation core for independent statistical analysis.

**Figure 3:**
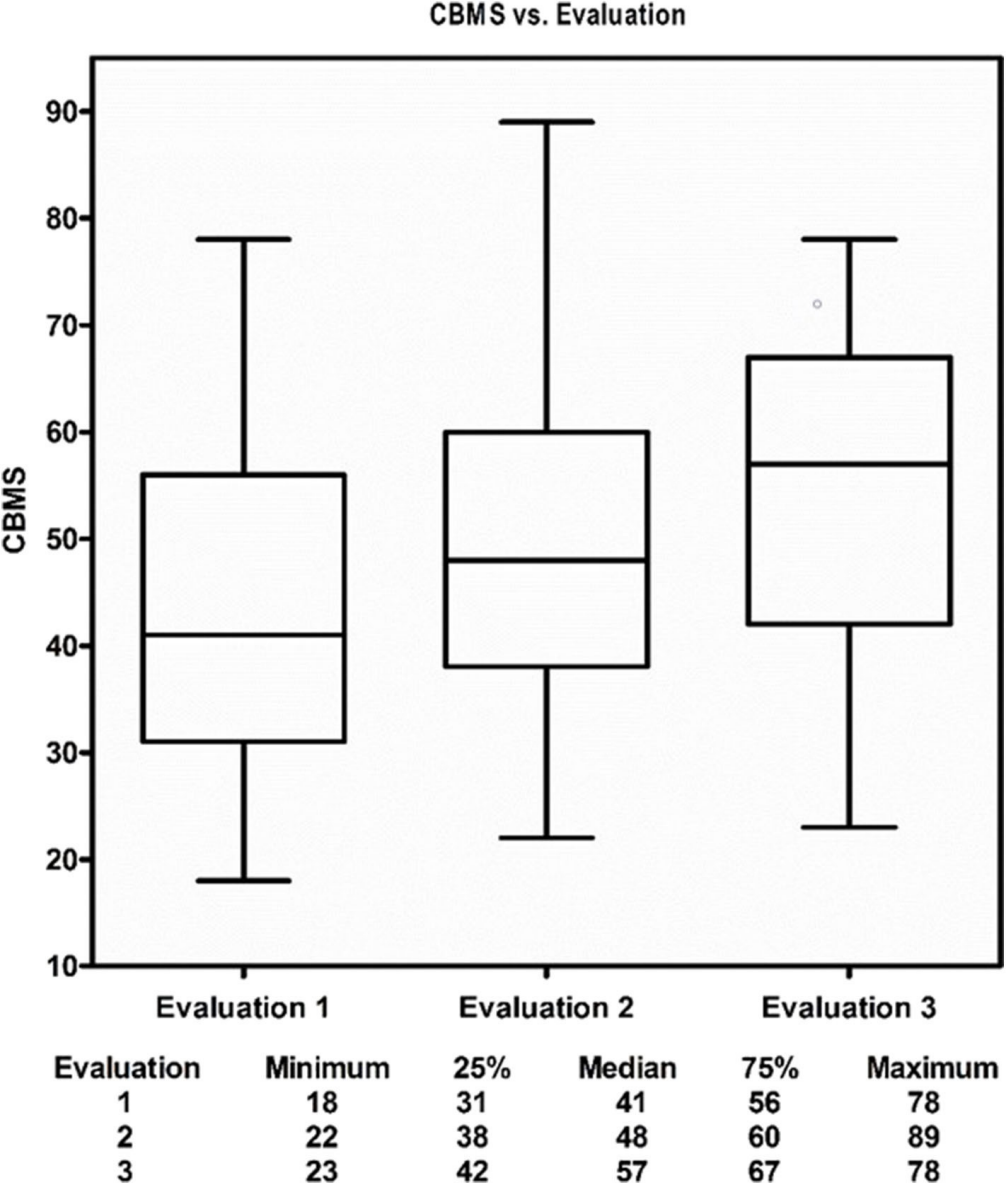
The Medical Qigong Intervention.

**Figure 4:**
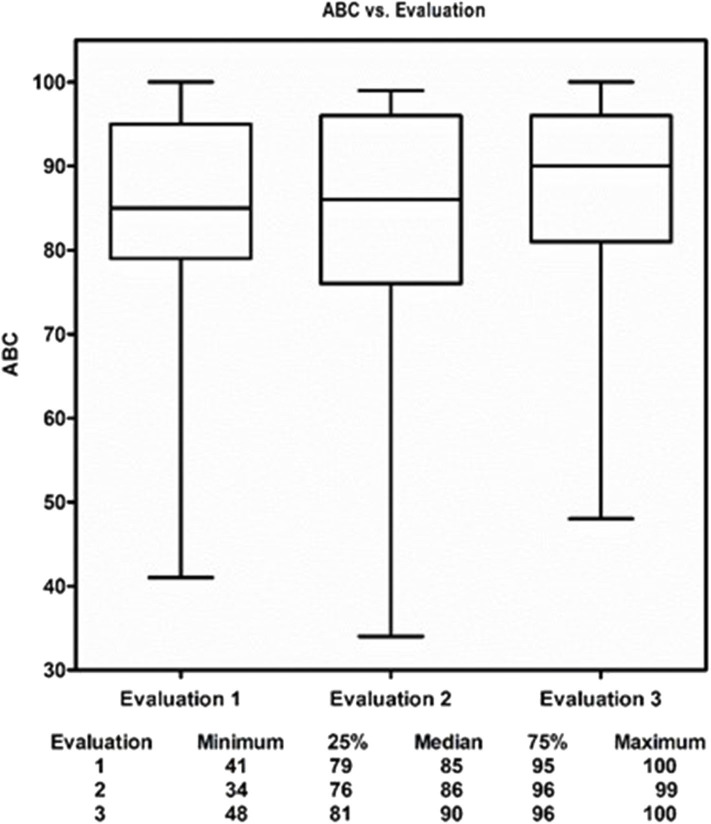
Change in subjects physical balance competence from pre-intervention to post-intervention.

Descriptive analysis of continuous variables included median and interquartile range (IQR), or mean and standard deviation (SD) as appropriate. Categorical variables were reported as counts and percentages. Baseline characteristics were compared between the two locations (Arizona vs. Massachusetts) using Chi-square test or Fisher's exact test where appropriate for categorical variables and *t*-test for continuous variables for all enrolled 95 participants (Table 1) and for the 75 participants who had at least one follow-up and were in the subsequent outcomes analyses (Appendix 1: Table 1). Of the 95 enrolled participants, 75 had at least one follow-up (evaluation 2 and/or evaluation 3) and were in the subsequent outcome analyses. Boxplots depicted descriptive statistics of CBMS and ABC at evaluations 1, 2, and 3 for the 75 participants who had at least one follow-up (Figures 3, 4). Direct cross-arm comparisons, e.g., t1 immediate start to Wait control t0 are presented in Table 2, for example, comparing the immediate start arm's outcome measures after the first month to its control, the wait time group after 1 month of education and stretching.

**Table 1:** Demographics for all enrolled participants.

	**ALL**	**Arizona**	**Massachusetts**	***P* value**
	**(*n* = 95)**	**(*n* = 42)**	**(*n* = 53)**	
Age (years),				<0.001
Mean (SD)	68.6 (9.1)	73.3 (9.1)	64.9 (7.4)	
Median (IQR)	68 (63, 76)	73.5 (67, 79)	65 (59, 70)	
Female, n (%)	80 (84%)	31 (74%)	49 (92%)	0.022
**Yoga**				
Current, n (%)	20 (21%)	1 (2%)	19 (36%)	<0.001
Ever, n (%)	42 (44%)	16 (38%)	26 (49%)	0.31
Years, median (IQR)	2 (1, 5)	4 (1, 15)	2 (0.67, 4.75)	
**TaiChi/martial arts**				
Current, n (%)	8 (8%)	2 (5%)	6 (11%)	0.30
Ever, n (%)	15 (16%)	7 (17%)	8 (15%)	1
Years, median (IQR)	1 (0.25, 2)	1.25 (0.17, 2)	1 (0.25, 2)	
**Meditation**				
Current, n (%)	29 (31%)	6 (14%)	23 (43%)	0.003
Ever, n (%)	15 (16%)	8 (19%)	7 (13%)	0.57
Years, median (IQR)	5 (1, 20)	3.5 (2, 20)	5 (1, 19)	
**Balance training**				
Current, n (%)	11 (12%)	7 (17%)	4 (8%)	0.21
Ever, n (%)	9 (9%)	5 (12%)	4 (8%)	0.50
Years, median (IQR)	0.5 (0.25, 3)	0.25 (0.13, 1)	2 (0.25, 5)	
**Gait training**				
Current, n (%)	7 (7%)	3 (7%)	4 (8%)	1
Ever, n (%)	18 (19%)	7 (17%)	11 (21%)	0.79
Assistive device, n (%)				<0.001
No	84 (88%)	32 (76%)	52 (98%)	
Sometimes	10 (11%)	10 (24%)	0 (0%)	
Yes	1 (1%)	0 (0%)	1 (2%)	
Current Med Dx re: mobility, n (%)	10 (11%)	9 (21%)	1 (2%)	0.004
Current Neurologic Dx re: balance, n (%)	13 (14%)	9 (21%)	4 (8%)	0.071
Injury within 1 yr. re: balance/mobility, n (%)	15 (16%)	7 (17%)	8 (15%)	1
Falls within 12 mos, n (%)	32 (34%)	16 (38%)	16 (30%)	0.51
number of falls, median (IQR)	2 (1, 3)	2 (1.5, 3.5)	2 (1, 2)	
Median Income ($K), median (IQR)	61 (46, 103)	46 (46, 50)	103 (61, 103)	<0.001
%HS or higher (%), median (IQR)	95 (91, 97)	95 (91, 95)	95 (9, 97)	93
Baseline ABC, median (IQR)	85 (78, 96)	80 (71, 87)	90 (83, 97)	<0.001
Baseline CBMS, median (IQR)	41 (31, 57)	39 (31, 59)	41 (32, 55)	0.40

*SD, indicates standard deviation; IQR, interquartile range (25–75%); ABC, Activities-Specific Balance Confidence scale; CBMS, Community Balance and Mobility Scale*.

**Table 2:** Immediate start vs. WT control: comparison at each t0, t1, and t2.

	**CBMS**					**ABC**				
	**Immediate**		**Wait**		***P* diff**.	**Immediate**		**Wait**		***P* diff**.
**Time**	T0	43.3		1 mos.		T0	86		1 mos.	
	T1	51.2	39.8	T0	0.0013	T1	83.3	83	T0	ns
	T2	57.1	46.7	T1	0.0034	T2	86.4	79.7	T1	0.16
			51.9	T2				80.3	T2	

*T0, intake evaluation; t1, first evaluation; t2, second evaluation*.

Predictors of change in scores as a whole compared with baseline for outcomes CBMS and ABC at each follow-up were selected via stepwise general linear models with an inclusion criterion of *P* < 0.1 to enter and *P* > 0.05 to exit, while age and gender were forced in the models (Appendix 2). Variables significant for outcomes CBMS and/or ABC at evaluation 2 or evaluation 3 were included as adjusting covariates in all mixed-effects longitudinal regression models. In addition, age, gender, location, and baseline outcome were included in all longitudinal outcome models. Outcomes analyses using changes from baseline at each follow-up, with a mixed-effects longitudinal regression model including a random individual effect to account for correlation between repeated measurements within individuals (Appendix 1: Tables 3a–c). The parameter estimates from the mixed-effects longitudinal regression models were provided in Appendix 2. Computations were performed using SAS procedure PROC MIXED (SAS version 9.4, SAS Institute Inc., Cary, NC). Statistical significance was defined as *p* < 0.05 based on a two-sided hypothesis test with no adjustments made for multiple comparisons. Sample size is an a priori best guess estimate of the number of subjects needed to detect a hypothesized difference. Based on being able to detect a 10% effect size difference between 2 means, assuming alpha = 0.05, b = 0.8 and an estimated standard deviation ranging from 10 to 20% our estimated sample size was between 34 and 128 with 73 as our intermediate working sample size (std. dev. = 15%) estimate. With a historically typical class/group size at these schools of 15-30, with 4 planned subject groups, this was felt to be an achievable goal.

## Results

A total of 95 participants were enrolled across the two sites (42 in AZ and 53 in MA; see Table 1). The median age for the group on enrollment was 68.6 (s.d. = 9.1) and included more women (84%) than men (16%). The Massachusetts group was statistically younger than the Arizona group [64.9 y.o. vs. 73.3 y.o. (*p* < 0.001)], and had a higher proportion of women [92% vs 74%, (*p* < 0.02)]. The Massachusetts group also had a higher median income ($103,000/year vs. $61,000/year, *p* < 0.05), but education levels as measured by high school graduation rates were statistically the same. A significant minority carried a medical (11%) and/or neurologic diagnosis (14%) that potentially impacts balance. Approximately one third of participants had fallen in the prior year. The Massachusetts groups were also able to access the dojos on non-class days - 10 in C and 12 in D took advantage. These were slightly older than the median 67 v 64.9 *p* = ns, were all female and had a slightly higher median income (81 K v 65 K) *p* < 0.05, and slightly lower estimated median HS graduation rate (0.92 v.93) *p* < 0.05). The wait groups, B and D, compared to the immediate start groups at each site were less likely to be currently engaged in yoga, Taichi or meditation at the time of the study and had lower initial ABC scores.

The majority of participants were not actively engaged in any other active mind-body practice (i.e., yoga, tai chi, gait training or meditation) that might have had an impact on outcomes. The majority of participants had tried meditation at some point in the past.

Thirty-eight people did not complete the program. Of these: 24% were lost to outside illness, 18% to outside injury, 16% due to family related issues, 16% couldn't attend due scheduling conflicts, 26% dropped out for unknown reasons. These differences were not statistically significant across geography or strata such as age decile.

The inter-rater agreement for evaluators of the CBMS score, i.e., Cohen's Kappa was 0.87. The observed agreement was 0.875. We assumed the likelihood of agreement by chance was 5%.

Both groups at both sites demonstrated improved balance and gait (CBMS + 11.9 points, *p* < 0.001). This effect was strongest in patients in their 60 s (CBMS +12.9 *p* < 0.01) and 70 s (CBMS + 14.3, *p* < 0.001), was equal across genders and socioeconomic status. Balance self-confidence did not significantly change (ABC + 0.9, *p* = 0.48), though several elements within ABC trended toward improvement [e.g., walk up/down ramp (*p* = 0.07), bend over/pick up (*p* = 0.09)]. Falls in the past year was inversely correlated with balance self-confidence (*p* = 0.01).

In univariate analyses, current martial arts and history of meditation were both predictors of improved performance on the CBMS. Prior falls, presence of a neurologic or medical diagnosis, or current use of assistive devices all predicted poorer performance on the CBMS. Participant CBMS performance steadily improved over the course of the study; there seemed to be a clear dose response relationship between the number of medical Qigong sessions attended and the improvements in CBMS scores as noted in the cross-arm analysis. Table 2 the improvements in ABC scores lagged behind the improvements in CBMS scores by ~1 month (Figures 3, 4).

Multivariate analysis revealed a best fit model to predict CBMS, with an R2 of 0.35 and for ABC R2 of 0.18. Candidate variables with most influence on regression for CBMS included subjects baseline scores and TaiChi exposure, for ABC these were exposure to therapy for gait and balance outside of the study and meditation (See Appendix 2).

There was no statistical difference in the CBMS and ABC scores between the Boston and Arizona sites at the same exposure level though the delayed start groups, Group 2, at both sites seemed have a higher baseline CBMS score (Appendix 1: Tables 3b,c).

## Discussion

The primary finding from the study was that participation in the 12-week manualized Qigong protocol as a primary intervention led to both statistically and clinically significant improvement in balance, mobility and health confidence. These findings are consistent with research on other forms of exercise, including Tai Chi, that have led to improvements in balance and mobility (9, 15). While exercise itself has been shown to reduce falls risk overall by 21% in community-dwelling older adults, the effect size increases to 39% if the program specifically challenges balance and includes 3 or more hours of exercise per week (12). The program studied appears to achieve similar outcomes in less time. Improved physical and mental function can be considered a proxy for reduced fall risk which implies significant savings of cost, morbidity and mortality.

Fear of falling is an important modifiable risk factor for falls. Fear can reduce quality of life and cause a feedback loop where fear of falling can cause activity restriction which reduces strength and balance which in turn increases fear (28). Therefore, building balance self-confidence should improve quality of life and mitigate the negative consequences of restricted activity, such as, reduced strength and muscle tone, attention deficits and diminished ability to perform complex locomotor tasks. In our study on average, ABC scores dipped slightly 1-month into treatment, only to regain and surpass baseline levels upon completion of the 3-month evaluation. There are many reasons why confidence levels may be temporarily reduced. Participants shared on feedback forms that Qigong practice helped them to see how much their fitness had deteriorated with time. Many found they were less physically capable than they originally thought and were therefore initially humbled by the training. At first, the awareness was disheartening, but when the practice was maintained, a new degree of mastery and confidence could emerge following the acquisition of new skills and levels of fitness.

Fear of falling and fear of loud noises are the two innate fears in mammals. Qigong, like tai chi, yoga, MBSR and other mind body approaches, that cultivate mindfulness and body awareness have also been shown to reduce psychological stress and anxiety (29–34). Thus, could have been at least in part the reduced fear of falling in addition to increased physical competency.

Participants at the Boston location were not restricted to one class per week. As such, some participants elected to come to class multiple times per week. This afforded an opportunity to conduct a dose-response analysis. As expected, more frequent practice led to more significant gains across measures.

Qigong was selected for this intervention for several reasons. It is a low-impact and gentle form of exercise that can be performed by individuals with a wide range of fitness levels and abilities. It works on the four main domains the equilibrium system. It develops and trains the sensory systems involved in balance and sense of position and internal state. It develops both static and dynamic strength and develops situational awareness and reactive strategies for disequilibrium and anticipatory strategies for movement. As a meditative practice, it also develops the insula, which is a center in the brain that focuses on sensing one's internal state, interoception, and the regulation of the body's homeostasis (35). These functions include compassion and empathy, perception, motor control, cognitive functioning, self-awareness and interpersonal experience.

Compared to Tai Chi, which emphasizes more complex movements for fitness and self-defense, the movements in Qigong are intended to cultivate vital energy (“Chi”) and improve well-being. Recent research found that a Qigong exercise program was met with high levels of acceptability and feasibility among community-dwelling older adults, with no adverse events, and with subjective reports of improved calm, relaxation, balance and flexibility (36). Qigong is also considered simpler and easier to teach than Tai Chi and more adaptable to a variety of body types and functional limits (17). Its ease of practice and simplicity make it a very good candidate as a primary intervention, as per the National Council on Aging's recommendations.

## Implications for Future Research

The findings from the study suggest the need for larger-scale randomized clinical trials powered to explore the effects of this intervention on different components of the CBMS and ABC scales and further explore the barriers and facilitators of a manualized medical Qigong protocol delivered in highly accessible community settings.

## Strengths and Limitations

The multi-site design was a strength for this study. This allowed us to identify any unusual trends or biases within each study group. In addition, the intervention being manualized is very reproducible allowing future comparative studies to be conducted.

A limitation of the study was the relatively high dropout rate which might bias the results. That said the rate and identified causes were consistent across groups and geographic locations suggesting no local selection biases were at play. Rather it might imply a more general reason which could have caused an unknown bias either in favor or against the results. For example, subjects that dropped because of illness might result in a bias in favor of the results or subjects leaving for scheduling, family or other unknown reasons, for example, perhaps finding the program not challenging enough or not moving fast enough might result a bias against the outcomes. In either instance, the results are consistent with other similar Tai Chi or Qigong interventions.

## Data Availability Statement

The raw data supporting the conclusions of this article will be made available by the authors, without undue reservation.

## Ethics Statement

The studies involving human participants were reviewed and approved by Committee for the Protection of Human Subjects at Dartmouth Hitchcock Medical Center and Dartmouth College. The patients/participants provided their written informed consent to participate in this study.

## Author Contributions

JS was responsible for study inception and design, recruitment of study sites, data interpretation, and manuscript preparation. SB assisted with data management, analysis and interpretation, literature review, drafting of manuscript, and preparation of manuscript for publication. WZ was responsible for the statistical analysis and interpretation of data. All authors contributed to the article and approved the submitted version.

## Conflict of Interest

The authors declare that the research was conducted in the absence of any commercial or financial relationships that could be construed as a potential conflict of interest.
