# Supplementary material for: Medical Qigong for Mobility and Balance Self-Confidence in Older Adults
Source: Front Med (Lausanne). 2020 Aug 14;7:422. doi: 10.3389/fmed.2020.00422 (PMC7456993; doi:10.3389/fmed.2020.00422)
Supplement: Supplementary file 1 [file Table_1.docx]

# Appendix 1

| **Table 1. Demographics for participants with at least one follow-up and in the subsequent outcomes analyses** | | | | |
| --- | --- | --- | --- | --- |
|  | **ALL** | **Arizona** | **Massachusetts** | ***P* value** |
|  | (n=75) | (n=33) | (n=42) |  |
| Age (years), |  |  |  | <0.001 |
| Mean (SD) | 68.9 (9.2) | 73.4 (9.6) | 65.4 (7.2) |  |
| Median (IQR) | 68 (63, 76) | 76 (66, 79) | 65.5 (62, 70) |  |
| Female, n (%) | 66 (88%) | 25 (76%) | 41 (98%) | 0.009 |
| Yoga  Current, n (%) | 16 (21%) | 1 (3%) | 15 (36%) | <0.001 |
| Ever, n (%) | 37 (49%) | 15 (45%) | 22 (52%) | 0.64 |
| Years, median (IQR) | 2 (0.75, 5) | 5 (1, 15) | 2 (0.58, 4) |  |
| TaiChi/ Martial Arts  Current, n (%) | 8 (11%) | 2 (6%) | 6 (14%) | 0.45 |
| Ever, n (%) | 14 (19%) | 7 (21%) | 7 (17%) | 0.77 |
| Years, median (IQR) | 0.79 (0.25, 2) | 1.25 (0.17, 2) | 0.79 (0.25, 2.5) |  |
| Meditation  Current, n (%) | 20 (27%) | 4 (12%) | 16 (38%) | 0.017 |
| Ever, n (%) | 11 (15%) | 7 (21%) | 4 (10%) | 0.20 |
| Years, median (IQR) | 7.5 (2, 20) | 2 (2, 5) | 15 (4, 25) |  |
| Balance training  Current, n (%) | 9 (12%) | 5 (15%) | 4 (10%) | 0.49 |
| Ever, n (%) | 9 (12%) | 5 (15%) | 4 (10%) | 0.49 |
| Years, median (IQR) | 0.38 (0.21, 3) | 0.21 (0.13, 0.5) | 2 (0.25, 5) |  |
| Gait training  Current, n (%) | 6 (8%) | 2 (6%) | 4 (10%) | 0.69 |
| Ever, n (%) | 14 (19%) | 6 (18%) | 8 (19%) | 1 |
| Assistive device, n (%) |  |  |  | 0.002 |
| No | 68 (91%) | 26 (79%) | 42 (100%) |  |
| Sometimes | 7 (9%) | 7 (21%) | 0 ( 0%) |  |
| Yes | 0 (0%) | 0 ( 0%) | 0 ( 0%) |  |
| Current Med Dx re: mobility, n (%) | 8 (11%) | 8 (24%) | 0 (0%) | <0.001 |
| Current Neurologic Dx re: balance, n (%) | 9 (12%) | 7 (21%) | 2 (5%) | 0.038 |
| Injury within 1 yr. re: balance/mobility, n (%) | 13 (17%) | 7 (21%) | 6 (14%) | 0.54 |
| Falls within 12mos, n (%) | 27 (36%) | 12 (36%) | 15 (36%) | 1 |
| # falls, median (IQR) | 2 (1, 2) | 2 (1.5, 3) | 2 (1, 2) |  |
| Median Income ($K), median (IQR) | 61 (46, 103) | 46 (46, 50) | 86 (61, 103) | <0.001 |
| %HS or higher (%), median (IQR) | 0.95 (0.91, 0.95) | 0.95 (0.93, 0.95) | 0.95 (0.9, 0.97) | 0.58 |
| Baseline ABC, median (IQR) | 85 (79, 95) | 82 (75, 88) | 90 (83, 96) | 0.003 |
| Baseline CBMS, median (IQR) | 41 (31, 56) | 37 (31, 59) | 41 (32, 54) | 0.39 |

SD indicates standard deviation; IQR, interquartile range (25%-75%); ABC, Activities-Specific Balance Confidence scale; CBMS, Community Balance and Mobility Scale

| Table 2. Demographics for each group | | | | | |
| --- | --- | --- | --- | --- | --- |
|  | **Arizona** |  | **Massachusetts** |  | ***ANOVA P* value** |
|  | Immediate (n=24) | Wait Start (n=18) | Immediate (n=28) | Wait Start (n=25) |  |
| Age (years), |  |  |  |  |  |
| Mean (SD)*ns within location | 73.7 | 73.2 | 64.1 | 66.1 | <.0001 |
| Median (IQR) | 74 | 76 | 64 | 67 |  |
| Female, n (%) | 65.4 | 86.4 | 92.4 | 86.7 | <.0001 |
| Yoga  Current, n (%) | 6.1 | 0 | 45 | 25 | <0.001 |
| Ever, n (%) | 50 | 56.4 | 73.7 | 67.2 | ns |
| Years, median (IQR) | 20( 1-30) | 3(1-10) | 2(.6-4) | 1.5(.5-5) |  |
| TaiChi/ Martial Arts  Current, n (%) | 13.3 | 0 | 19 | 9.4 | 0.03 |
| Ever, n (%) | 33.3 | 34.8 | 29.4 | 29.8 | ns |
| Years, median (IQR) | 1.1(.1-11.8) | 1.25(.5-2) | 1(.6-3) | .25(.17-1) |  |
| Meditation  Current, n (%) | 20.5 | 5.7 | 47 | 37.3 | <.0001 |
| Ever, n (%) | 31 | 26.1 | 18.2 | 57.1 | 0.07 |
| Years, median (IQR) | 3.5(2-20) | 1(1-1) | 19(4-30) | 1(.6-4) |  |
| Gait and Balance training  Current, n (%) | 8 | 7.3 | 7.6 | 19.4 | ns |
| Ever, n (%) | 6.7 | 38.9 | 13.9 | 45.6 | <.0001 |
| Years, median (IQR) | 1(1-1) | .17(.12-.5) | 3(.8-5.8) | .25(.25-.25) |  |
| Assistive device, n (%) |  |  |  |  | <0.001 |
| No | 84.6 | 70.5 | 100 | 98.4 |  |
| Sometimes | 15.4 | 29.5 | 0 | 0 |  |
| Yes | 0 | 0 | 0 | 1.6 |  |
| Current Med Dx re: mobility, n (%) | 22.4 | 25 | 1.5 | 0 | <.001 |
| Current Neurologic Dx re: balance, n (%) | 17.6 | 25 | 3 | 9.5 | 0.08 |
| Injury within 1 yr. re: balance/mobility, n (%) | 17.3 | 20.5 | 19.7 | 11.5 | .6 |
| Falls within 12mos, n (%) | 28.9 | 50 | 31.8 | 31.3 | 0..12 |
| # falls, median (IQR) | 2(1-2) | 2(2-5) | 2(1-4) | 2(1-2) |  |
| Median Income ($K), median (IQR) | 46 K(46-67) | 46(46-46) | 103(61-103) | 80(61-103) | <0.001 |
| %HS or higher (%), median (IQR) | .95(.91-.95) | .95(.91-.95) | .97(.9-.97) | .9(.91-.95) | 0.79 |
| Baseline ABC, median (IQR) | 82.8(72-87.3) | 76.9(66.3-86.6) | 93.7(83.3-97.5) | 87.5(82.6-96.4) | <0.001 |
| Baseline CBMS, median (IQR) | 47.6(31.6-57.9) | 33.6(30.6-64.1) | 48.9(33.1-59.7) | 38.9(29.4-57.3) | 0.2 |

SD indicates standard deviation; IQR, interquartile range (25%-75%); ABC, Activities-Specific Balance

**Table 3a. Change in scores compared with baseline for outcomes ABC and CBMS*†**

|  | Baseline (t0) | Evaluation (t1) | | Evaluation (t2) | |
| --- | --- | --- | --- | --- | --- |
|  | Overall Mean  (SE) | Mean change in score compared with baseline (SE) | *P* value | Mean change in score compared with baseline (SE) | *P* value |
|  | (n = 75) | (n = 75) |  | (n = 57) |  |
| ABC | 84.8 (1.4) | -1.5 (1.1) | 0.19 | 0.9 (1.2) | 0.48 |
| CBMS | 43.3 (1.7) | 5.3 (0.8) | **<0.001** | 11.9 (0.9) | **<0.001** |

*Scores are adjusted for age, gender, current TaiChi/ Martial Arts, current meditation, current gait training, group, baseline score (for ABC and CBMS), and location.

†Seventy-five participants who had at least one follow-up were in the outcome analysis with the use of mixed-effects longitudinal regression model.

SE indicates standard error; ABC, Activities-Specific Balance Confidence Scale; CBMS, Community Balance and Mobility Scale.

**Table 3b. Change scores and their differences ('group 1' minus 'group 2') for outcomes ABC and CBMS, according to group*†**

|  | Baseline (t0) | Evaluation (t1) | | | Evaluation (t2) | | |  |
| --- | --- | --- | --- | --- | --- | --- | --- | --- |
|  | Overall Mean | Mean change in score compared with baseline  (SE) | | | Mean change in score compared with baseline  (SE) | | |  |
|  |  | Group 1 | Group 2 | *P* value | Group 1 | Group 2 | *P* value |  |
|  | (n = 75) | (n = 37) | (n = 38) |  | (n = 29) | (n = 28) |  |  |
| ABC | 84.8 (1.4) | -2 (1.6) | -0.9 (1.6) | 0.64 | 1.3 (1.7) | 0.4 (1.9) | 0.75 |  |
| CBMS | 43.3 (1.7) | 3.5 (1.2) | 7.2 (1.2) | **0.035** | 9.8 (1.3) | 14 (1.3) | **0.026** |  |

*Scores are adjusted for age, gender, current TaiChi/ Martial Arts, current meditation, current gait training, group, baseline score (for ABC and CBMS), and location.

†Seventy-five participants who had at least one follow-up were in the outcome analysis with the use of mixed-effects longitudinal regression model.

SE indicates standard error; ABC, Activities-Specific Balance Confidence Scale; CBMS, Community Balance and Mobility Scale.

**Table 3c. Change scores and their differences ("Arizona" minus "Massachusetts") for outcomes ABC and CBMS, according to location*†**

|  | Baseline overall  mean (SE) | Evaluation 2 | | |  | Evaluation 3 | | |  |
| --- | --- | --- | --- | --- | --- | --- | --- | --- | --- |
|  |  | Mean change in score compared with baseline (SE) | | Difference (95% CI) | *P* value | Mean change in score compared with baseline (SE) | | Difference (95% CI) | *P* value |
|  |  | AZ | MA |  |  | AZ | MA |  |  |
|  | (n = 75) | (n = 33) | (n = 42) |  |  | (n = 21) | (n = 36) |  |  |
| ABC | 84.8 (1.4) | -1.4 (2.1) | -1.5 (1.6) | 0.1 (-5.6, 5.7) | 0.99 | 2.1 (2.4) | 0.2 (1.7) | 1.9 (-4.4, 8.1) | 0.56 |
| CBMS | 43.3 (1.7) | 4.3 (1.6) | 5.8 (1.2) | -1.5 (-5.9, 2.9) | 0.50 | 8.9 (1.8) | 13.3 (1.2) | -4.4 (-9.1, 0.3) | 0.073 |

*Scores are adjusted for age, gender, current TaiChi/ Martial Arts, current meditation, current gait training, group, baseline score (for ABC and CBMS), and location.

†Seventy-five participants who had at least one follow-up were in the outcome analysis with the use of mixed-effects longitudinal regression model.

SE indicates standard error; ABC, Activities-Specific Balance Confidence Scale; CBMS, Community Balance and Mobility Scale.
