# Supplementary material for: Medical Qigong for Mobility and Balance Self-Confidence in Older Adults
Source: Front Med (Lausanne). 2020 Aug 14;7:422. doi: 10.3389/fmed.2020.00422 (PMC7456993; doi:10.3389/fmed.2020.00422)
Supplement: Supplementary file 2 [file Table_2.docx]

Appendix 2

| **General linear regression models: variable selection results for outcomes CBMS and ABC (change in scores) at evaluations 2 and 3, respectively*** | | | | | | | | | | | | | | | | |
| --- | --- | --- | --- | --- | --- | --- | --- | --- | --- | --- | --- | --- | --- | --- | --- | --- |
|  |  |  |  |  |  |  |  |  |  |  |  |  |  |  |  |  |
| **Outcome** | | **Eval #** | **Effect** | | **Forced-In** | | **DF** | **Estimate** | | **Standard Error** | | | **tValue** | | ***P* value** | |
| **CBMS** | | 2 | Intercept | | 0 | | 1 | 31.83006 | | 11.346838 | | | 2.81 | | 0.007 | |
| **(R^2^ = 0.35)** | | 2 | Age | | 1 | | 1 | -0.1014 | | 0.110243 | | | -0.92 | | 0.3618 | |
|  | | 2 | Gender F | | 1 | | 1 | -2.1215 | | 2.838793 | | | -0.75 | | 0.4582 | |
|  | | 2 | Gender M | | 1 | | 0 | 0 | |  | | |  | |  | |
|  | | 2 | Baseline CBM_AdjTotal | | 0 | | 1 | -0.22346 | | 0.067532 | | | -3.31 | | **0.0017** | |
|  | | 2 | Group 1 | | 0 | | 1 | -3.7125 | | 1.635298 | | | -2.27 | | **0.0273** | |
|  | | 2 | Group 2 | | 0 | | 0 | 0 | |  | | |  | |  | |
|  | | 2 | Current TaiChi/ Martial Arts N | | 0 | | 1 | -7.70095 | | 2.520896 | | | -3.05 | | **0.0035** | |
|  | | 2 | Current TaiChi/ Martial Arts Y | | 0 | | 0 | 0 | |  | | |  | |  | |
| **CBMS** | | 3 | Intercept | | 0 | | 1 | -1.0232 | | 9.371613 | | | -0.11 | | 0.9136 | |
| **(R^2^ = 0.36)** | | 3 | Age | | 1 | | 1 | 0.294312 | | 0.132429 | | | 2.22 | | **0.0318** | |
|  | | 3 | Gender F | | 1 | | 1 | -2.05209 | | 3.754488 | | | -0.55 | | 0.5876 | |
|  | | 3 | Gender M | | 1 | | 0 | 0 | |  | | |  | |  | |
|  | | 3 | Group 1 | | 0 | | 1 | -4.31833 | | 1.993225 | | | -2.17 | | **0.0361** | |
|  | | 3 | Group 2 | | 0 | | 0 | 0 | |  | | |  | |  | |
|  | | 3 | Location A | | 0 | | 1 | -10.948 | | 2.753905 | | | -3.98 | | **0.0003** | |
|  | | 3 | Location B | | 0 | | 0 | 0 | |  | | |  | |  | |
| **ABC** | | 2 | Intercept | | 0 | | 1 | -6.30604 | | 11.32055 | | | -0.56 | | 0.5798 | |
| **(R^2^ = 0.18)** | | 2 | Age | | 1 | | 1 | -0.05585 | | 0.129713 | | | -0.43 | | 0.6685 | |
|  | | 2 | Gender F | | 1 | | 1 | 1.884825 | | 3.309162 | | | 0.57 | | 0.5714 | |
|  | | 2 | Gender M | | 1 | | 0 | 0 | |  | | |  | |  | |
|  | | 2 | Current Meditation N | | 0 | | 1 | -5.09358 | | 2.37629 | | | -2.14 | | **0.0367** | |
|  | | 2 | Current Meditation Y | | 0 | | 0 | 0 | |  | | |  | |  | |
|  | | 2 | Current Gait & Balance Tx N | | 0 | | 1 | 11.06214 | | 4.29518 | | | 2.58 | | **0.0128** | |
|  | | 2 | Current Gait & Balance Tx Y | | 0 | | 0 | 0 | |  | | |  | |  | |
| **ABC** | | 3 | Intercept | | 0 | | 1 | 34.48437 | | 14.036192 | | | 2.46 | | 0.0183 | |
| **(R^2^ = 0.17)** | | 3 | Age | | 1 | | 1 | -0.15279 | | 0.134218 | | | -1.14 | | 0.2616 | |
|  | | 3 | Gender F | | 1 | | 1 | -0.06287 | | 3.47146 | | | -0.02 | | 0.9856 | |
|  | | 3 | Gender M | | 1 | | 0 | 0 | |  | | |  | |  | |
|  | | 3 | Baseline ABC_Avg | | 0 | | 1 | -0.25393 | | 0.090085 | | | -2.82 | | **0.0074** | |
| *Variables were selected via stepwise general linear regression models with an inclusion criterion of P < 0.1 to enter and P > 0.05 to exit at evaluation 2 and evaluation 3, respectively, while age and gender were forced in the models from the candidate variables: age, gender, group, location, income, education, baseline outcome, current yoga, current TaiChi/Martial Arts, current meditation, current balance training, current gait training, assistive device, Current Med Dx re: mobility, Current Neurologic Dx re: balance, Injury within 1 yr. re: balance/mobility, Falls within 12 months. | | | | | | | | | | | | | | | | |
|  |  |  |  |  |  |  |  |  |  |  |  |  |  |  |  |  |
|  |  |  |  |  |  |  |  |  |  |  |  |  |  |  |  |  |
|  |  |  |  |  |  |  |  |  |  |  |  |  |  |  |  |  |
| Note: past yoga, past TaiChi/Martial Arts, past meditation, past balance training, and past gait training were not included in the candidate predictor list because of missing data. | | | | | | | | | | | | | | | | |
|  |  |  |  |  |  |  |  |  |  |  |  |  |  |  |  |  |
| **Mixed-effects longitudinal regression model: parameters estimates** | | | | | | | | | | |  |  | |  | |  |
| **Outcome** | **Effect** | | | **Level** | | **Estimate** | | | **Standard Error** | | **DF** | **tValue** | | ***P* value** | |  |
| **CBMS** | Intercept | | |  | | 30.7289 | | | 12.1687 | | 56 | 2.53 | | 0.0144 | |  |
|  | Baseline CBM_AdjTotal | | |  | | -0.1624 | | | 0.06789 | | 47 | -2.39 | | **0.0208** | |  |
|  | Location | | | A | | -2.5587 | | | 2.186 | | 47 | -1.17 | | 0.2477 | |  |
|  | Location | | | B | | 0 | | |  | |  |  | |  | |  |
|  | Age | | |  | | 0.006134 | | | 0.1216 | | 47 | 0.05 | | 0.96 | |  |
|  | Gender | | | F | | -1.5525 | | | 2.9506 | | 47 | -0.53 | | 0.6012 | |  |
|  | Gender | | | M | | 0 | | |  | |  |  | |  | |  |
|  | Current Meditation | | | N | | 0.3547 | | | 1.7969 | | 47 | 0.2 | | 0.8444 | |  |
|  | Current Meditation | | | Y | | 0 | | |  | |  |  | |  | |  |
|  | Current Gait & Balance Tx | | | N | | -0.9764 | | | 2.9582 | | 47 | -0.33 | | 0.7428 | |  |
|  | Current Gait & Balance Tx | | | Y | | 0 | | |  | |  |  | |  | |  |
|  | Current TaiChi/ Martial Arts | | | N | | -8.4098 | | | 2.4268 | | 47 | -3.47 | | **0.0011** | |  |
|  | Current TaiChi/ Martial Arts | | | Y | | 0 | | |  | |  |  | |  | |  |
|  | Group | | | 1 | | -4.2729 | | | 1.8552 | | 47 | -2.3 | | **0.0257** | |  |
|  | Group | | | 2 | | 0 | | |  | |  |  | |  | |  |
|  | Eval # | | | 2 | | -6.8078 | | | 1.1304 | | 47 | -6.02 | | **<.0001** | |  |
|  | Eval # | | | 3 | | 0 | | |  | |  |  | |  | |  |
|  | Group*Eval (interaction) | | | Group 1, Eval 2 | | 0.5045 | | | 1.5608 | | 47 | 0.32 | | 0.7479 | |  |
|  | Group*Eval (interaction) | | | Group 1, Eval 3 | | 0 | | |  | |  |  | |  | |  |
|  | Group*Eval (interaction) | | | Group 2, Eval 2 | | 0 | | |  | |  |  | |  | |  |
|  | Group*Eval (interaction) | | | Group 2, Eval 3 | | 0 | | |  | |  |  | |  | |  |
| **ABC** | Intercept | | |  | | 15.6413 | | | 14.173 | | 55 | 1.1 | | 0.2746 | |  |
|  | Baseline ABC_Avg | | |  | | -0.1722 | | | 0.08223 | | 46 | -2.09 | | **0.0418** | |  |
|  | Location | | | A | | 0.7216 | | | 2.5465 | | 46 | 0.28 | | 0.7782 | |  |
|  | Location | | | B | | 0 | | |  | |  |  | |  | |  |
|  | Age | | |  | | -0.08793 | | | 0.1246 | | 46 | -0.71 | | 0.4839 | |  |
|  | Gender | | | F | | 0.6706 | | | 3.2766 | | 46 | 0.2 | | 0.8387 | |  |
|  | Gender | | | M | | 0 | | |  | |  |  | |  | |  |
|  | Current Meditation | | | N | | -3.881 | | | 2.0848 | | 46 | -1.86 | | 0.0691 | |  |
|  | Current Meditation | | | Y | | 0 | | |  | |  |  | |  | |  |
|  | Current Gait & Balance Tx | | | N | | 8.0679 | | | 3.3975 | | 46 | 2.37 | | **0.0218** | |  |
|  | Current Gait & Balance Tx | | | Y | | 0 | | |  | |  |  | |  | |  |
|  | Current TaiChi/ Martial Arts | | | N | | 0.1051 | | | 2.7348 | | 46 | 0.04 | | 0.9695 | |  |
|  | Current TaiChi/ Martial Arts | | | Y | | 0 | | |  | |  |  | |  | |  |
|  | Group | | | 1 | | 0.8472 | | | 2.5894 | | 46 | 0.33 | | 0.745 | |  |
|  | Group | | | 2 | | 0 | | |  | |  |  | |  | |  |
|  | Eval # | | | 2 | | -1.3522 | | | 2.2398 | | 46 | -0.6 | | 0.549 | |  |
|  | Eval # | | | 3 | | 0 | | |  | |  |  | |  | |  |
|  | Group*Eval (interaction) | | | Group 1, Eval 2 | | -1.9312 | | | 3.1102 | | 46 | -0.62 | | 0.5377 | |  |
|  | Group*Eval (interaction) | | | Group 1, Eval 3 | | 0 | | |  | |  |  | |  | |  |
|  | Group*Eval (interaction) | | | Group 2, Eval 2 | | 0 | | |  | |  |  | |  | |  |
|  | Group*Eval (interaction) | | | Group 2, Eval 3 | | 0 | | |  | |  |  | |  | |  |
